# Immune dysfunction leads to mortality and organ injury in patients with COVID-19 in China: insights from ERS-COVID-19 study

**DOI:** 10.1038/s41392-020-0163-5

**Published:** 2020-05-05

**Authors:** Dongze Li, You Chen, Hong Liu, Yu Jia, Fanghui Li, Wei Wang, Jiang Wu, Zhi Wan, Yu Cao, Rui Zeng

**Affiliations:** 1 Department of Emergency Medicine and Laboratory of Emergency Medicine, Deep Underground Space Medical Center, Disaster Medical Center, West China School of Nursing, West China Hospital, Sichuan University, Chengdu, China; 2 Department of Cardiology, The First Affiliated Hospital of Xinjiang Medical University, Urumqi, China; 3 Department of Cardiology, West China Hospital, Sichuan University, Chengdu, China

**Keywords:** COVID-19, Immune dysfunction, Mortality, Organ injury, Infectious diseases, Immunological disorders

**Dear Editor,**

A series of novel coronavirus disease 2019 (COVID-19) caused by severe acute respiratory syndrome coronavirus 2 (SARS-CoV-2) since the end of 2019 is ongoing and triggering a global public health crisis. The estimated case fatality rate is approximately 3.4% in China. However, some patients experience dyspnea within 1 week and develop rapidly to organ injury and even death within 2 weeks after dyspnea.^1^ In addition, early organ injury could lead to higher risks of mortality. Thus, early identification of patients at risk of organ injury and death is crucial, which saves the patients from classified and invasive treatment, improving clinical outcome and prognosis. The human immune system plays significant roles in the resistance of foreign pathogens and the progress of pneumonia. Recent studies have mentioned that T cells were decreased in COVID-19 patients, excessive activated immune response was caused by pathogenic Th1 cells, and inflammatory CD14^+^CD16^+^ monocytes may connect to pulmonary immunopathology, leading to deleterious clinical manifestations and even acute mortality after SARS-CoV-2 infections.^2^ SARS-CoV-2 might damage lymphocytes, especially T lymphocytes, and the immune system was impaired during the period of disease to cause tissue injury.^2,3^

Therefore, immune dysfunction is very likely to be a risk factor for patients with COVID-19, and immunological profiling may assist in the prediction of organ injury and prognosis in COVID-19 patients. However, few studies have systematically reported immunological characteristics and their relationship with organ injury and mortality in patients with COVID-19.

In this multicenter retrospective cohort study, we analyzed data from the Early Risk Stratification of Novel Coronavirus Pneumonia (ERS-COVID-19) study. The study was registered at www.chictr.org.cn (Identifier: ChiCTR2000030494). The study complied with the Declaration of Helsinki, and the Human Ethical Committee of West China Hospital of Sichuan University approved the study protocol.

From January 31, 2020 to February 18, 2020, 509 patients retrospectively enrolled in the ERS-COVID-19 study. The COVID-19 was confirmed according to the National Health Commission of the People’s Republic of China and the National Administration of Traditional Chinese Medicine.^4^ The inclusion criteria were age >18 years old and first diagnosis of COVID-19. The exclusion criteria were examination without immune-related indicators before treatment, pregnancy, taking immunosuppressive drugs or corticosteroids, a history of chronic organ dysfunction or immunological disease, operation history within 3 months, and simultaneous infection with other diseases. Finally, a total of 163 patients were recruited in this study. The primary endpoint was all-cause death, and the secondary endpoint was MODS and severe pneumonia. All patients were followed up to 30 days after admission to the hospital.

Serum humoral immunity levels, such as immunoglobulin G (IgG), IgM, complement 3 (C3), and C4, were measured by immune rate nephelometry (Beckman Coulter IMMAGE^®^ 800 Immunochemistry System, Beckman Coulter Ireland, Inc., Brea, CA). The levels of T lymphocytes (CD3^+^, CD4^+^, and CD8^+^), B lymphocytes (CD19^+^), and natural killer (NK) cells (CD16^+^ CD56^+^) were detected by flow cytometry (six-color flow cytometry, BD Company, USA).

Of 163 patients with COVID-19, 66 (40.5%) patients had severe pneumonia, 25 (15.3%) patients combined pneumonia with MODS, and 27 (16.6%) patients died after they were hospitalized. In those patients, 33 (20.2%), 9 (5.5%), and 6 (3.7%) patients developed to acute lung injury, myocardial injury, and kidney injury, respectively. Table S1 shows the baseline characteristics of the patients divided into the survival and death groups.

In total, 113 (69.3%) patients had abnormal cellular immunity, and 58 (35.6%) had abnormal humoral immunity. Patients with abnormal cellular immunity had higher mortality, MODS, and severe pneumonia (*P* < 0.001). In contrast, those patients with abnormal humoral immunity only had higher mortality (*P* = 0.045, Table S2).

Patients with COVID-19 had elevated neutrophil, monocyte, high-sensitivity C-reactive protein (Hs-CRP), and procalcitonin, and showed decreased eosinophil, lymphocyte numbers, lymphocyte-immune subsets, IgM, and C3. Compared with survivors, the dead patients had a lower percentage of lymphocytes (*P* < 0.001), lymphocyte count (*P* < 0.001), CD3^+^ T-cell percentage (*P* < 0.001), CD3^+^ T-cell count (*P* < 0.001), CD4^+^ T-cell percentage (*P* = 0.013), CD4^+^ T-cell count (*P* < 0.001), CD8^+^ T-cell percentage (*P* = 0.002), CD8^+^ T-cell count (*P* < 0.001), and CD16^+^CD56^+^ NK-cell percentage (*P* < 0.001), whereas those patients had higher levels of leukocyte, neutrophil, complement 4, Hs-CRP, and procalcitonin in serum (*P* < 0.001, Table S3).

After adjusting for confounding factors, leukocyte count, neutrophil count, neutrophil percentage, lymphocyte percentage, complement 4, CD3^+^ T-cell percentage, CD3^+^ T-cell count, CD4^+^ T-cell count, CD8^+^ T-cell percentage, CD8^+^ T-cell count, and Hs-CRP remain independent prognostic factors. In addition, adjusted multivariate logistic regression model for MODS, leukocyte count, neutrophil count, neutrophil percentage, monocyte percentage, basophil percentage, basophil count, lymphocyte percentage, C3, C4, and CD3^+^ T-cell percentage, CD3^+^ T-cell count, CD4^+^ T- cell percentage, CD4^+^ T-cell count, and CD16^+^CD56^+^ NK-cell percentage were independent risk factors (Table S4).

In the ROC curve analysis, leukocyte count, neutrophil count, neutrophil percentage, monocyte percentage, basophil percentage, eosinophil count, eosinophil percentage, lymphocyte percentage, lymphocyte count, C4 and CD3^+^ T-cell percentage, CD3^+^ T-cell count, CD4^+^ T-cell percentage, CD4^+^ T-cell count, CD8^+^ T-cell percentage, CD8^+^ T-cell count, CD16^+^CD56^+^ NK-cell percentage, Hs-CRP, and procalcitonin had significant predictive power for in-hospital mortality (*P* < 0.05, Fig. 1a). Similarly, this immunological profiling had also significant predictive power for MODS (*P* < 0.05, Fig. 1b). Leukocyte count, neutrophil, lymphocytes, namely the CD3^+^, CD4^+^, and CD8^+^ T subsets, C4, Hs-CRP, and procalcitonin showed significant prediction efficiency for severe pneumonia (Fig. S1), acute lung injury (Fig. S2), myocardial injury (Fig. S3), and kidney injury (Fig. S4) in patients with COVID-19 (*P* < 0.05).

**Fig. 1:**
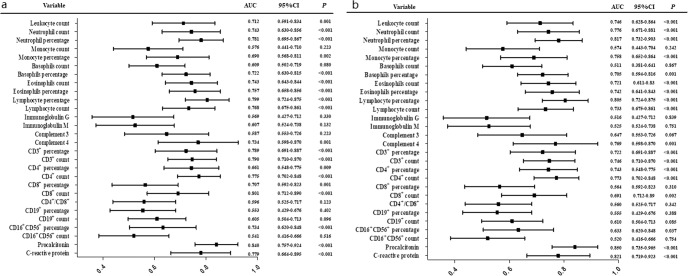
**a** Discrimination of immunological profiling for mortality. **b** Discrimination of immunological profiling for multiple organ dysfunction syndromes

In this study, we found that COVID-19 pneumonia manifests with immune dysfunction, even in patients with mild pneumonia. The patient’s leukocyte, neutrophil, lymphocyte counts, CD3^+^, CD4^+^, and CD8^+^ T-cell counts, C4, Hs-CRP, and procalcitonin had strong predictive values for in-hospital mortality, organ injury, and severe pneumonia. Decreased lymphocyte subsets and increased neutrophil, C4, and Hs-CRP were independently associated with high risks of mortality and organ injury, respectively. Therefore, clarifying the immunophenotype of COVID-19 pneumonia may contribute to the development of specific immunotherapy to correct the immune changes that lead to poor prognosis in patients with the disease.

## Supplementary information

Materials and Methods, Tables S1 to S4

Figure S1

Figure S2

Figure S3

Figure S4
