# Supplementary material for: Immune dysfunction leads to mortality and organ injury in patients with COVID-19 in China: insights from ERS-COVID-19 study
Source: Signal Transduct Target Ther. 2020 May 5;5:62. doi: 10.1038/s41392-020-0163-5 (PMC7198844; doi:10.1038/s41392-020-0163-5)
Supplement: Supplementary file 1 — Materials and Methods, Tables S1 to S4 [file 41392_2020_163_MOESM1_ESM.docx]

Supplementary Materials for

Immune dysfunction leads to mortality and organ injury in patients with COVID-19 in China: insights from ERS-COVID-19 study

Dongze Li, MBBS;^*^ You Chen, MD;^*^ Hong Liu, MD;^*^ Yu Jia, MD; Fanghui Li, MBBS; Wei Wang, MBBS; Jiang Wu, PhD; Zhi Wan, MD; Yu Cao, MD; and Rui Zeng, MD

^*^These authors contributed equally to this work.

Correspondence to: Prof. Rui Zeng, MD, E-mail: zengrui_0524@126.com

**This PDF file includes:**

Materials and Methods

Supplementary Text

Figures. S1 to S4

Tables S1 to S4

**Other Supplementary Materials for this manuscript include the following:**

None

Materials and Methods

**Study design**

In this multicenter retrospective cohort study, we analyzed data from the Early Risk Stratification of Novel Coronavirus Pneumonia (ERS-COVID-19) study to evaluate whether immunological profiling is associated with mortality in COVID-19 patients. The ERS-COVID-19 study enrolled in-hospital patients from two hospitals in Wuhan and one hospital in Chengdu, China. The study was registered at www.chictr.org.cn (Identifier: ChiCTR2000030494). The study complied with the Declaration of Helsinki, and the Human Ethical Committee of West China Hospital of Sichuan University approved the study protocol.

**Study population**

From January 31, 2020, to February 18, 2020, 509 patients retrospectively enrolled in the ERS-COVID-19 study. The diagnostic criterion for the study was testing positive with novel coronavirus RNA, by real-time reverse transcription-polymerase chain reaction or gene sequencing, from the sputum, throat swab, lower respiratory tract secretion, or other samples according to National Health Commission of the People’s Republic of China and the National Administration of Traditional Chinese Medicine (20). The inclusion criteria were: age >18 years old; first diagnosis of COVID-19. The exclusion criteria were: examination without immune-related indicators before treatment; pregnancy; taking immunosuppressive drugs or corticosteroids; a history of chronic organ dysfunction or immunological disease; operation history within three months; simultaneous infection with other diseases. Finally, a total of 163 patients were recruited in this study.

**Data collection**

We obtained data from the ERS-COVID-19 study. The trained physicians collected demographic information, vital signs, laboratory examinations, imaging, and adverse outcome according to electronic medical records. The lymphocyte numbers were analyzed using a hematology analysis system (LH750, Beckman Coulter Inc., Brea, CA). Serum humoral immunity levels, such as immunoglobulin G (IgG), IgM, complement 3 (C3), and C4, were measured by immune rate nephelometry (BNP specific protein analyzer, Siemens, Germany). The levels of T lymphocytes (CD3^+^, CD4^+^, and CD8^+^), B lymphocytes (CD19^+^), and Natural killer (NK) cells (CD16^+^ CD56^+^), were detected by flow cytometry (six-color flow cytometry, BD Company, USA).

**Definition**

The definition of severe pneumonia was as follows: respiratory rate ≥ 30 times/min; in the resting state, the oxygen saturation was ≤ 93%; partial pressure of oxygen (PaO_2_) / fraction of inspired oxygen (FiO_2_) ≤ 300 mmHg (20). MODS is defined as the acute and potentially reversible dysfunction of two or more organ systems, including cardiovascular, hepatobiliary, respiratory, neurological, hematological, gastrointestinal, and renal systems (21). Definition of acute kidney injury: serum creatinine increased by ≥ 0.3 mg/dl within 48 hours, or serum creatinine increased by ≥ 1.5 times the baseline value within seven days of disease occurrence, or if urine volume for six hours was < 0.5 ml/(kgh) (22, 23). Myocardial injury was identified by the upper reference limit for high sensitive troponin I (hs-TnI) test based on the 99th percentile of measurements reported in healthy men and women according to the manufacturer’s specification (24). Quantification of acute lung injury was carried out as PaO_2_/FiO_2_ ≤ 200 mmHg according to the criteria of acute respiratory distress syndrome (25).

**Endpoint**

The primary endpoint was all-cause death, and the secondary endpoint was MODS and severe pneumonia. Two trained doctors recorded all reported events, and a third member judged any disagreement between them. All patients were followed up to 30 days after admission to hospital.

**Statistical analysis**

The continuous variables were presented as means ± standard deviations or medians and interquartile ranges according to the distribution type of variables and compared using analysis of variance and Mann-Whitney U test, respectively. The categorical variables were presented as frequencies (percentages) and compared using the Chi-square test. A univariate and multivariate logistic regression model was employed to identify whether the variables are related to mortality and MODS. Multiple immune-related indicators established receiver operating characteristic curves (ROC), and the area under the curve (AUC) was used to evaluate the prognosis assessment ability. The correlation between immune indices and organ damage indexes was analyzed by Pearson or Spearman test. A two-sided *P* < 0.05 was considered significant. Data were analyzed using SPSS Statistics version 20.0 (Inc., Chicago, IL).

Supplementary Text

**Figures**

Figure. S1.

Discrimination of immunological profiling for severe pneumonia.

Figure. S2.

Discrimination of immunological profiling for acute lung injury.

Figure. S3.

Discrimination of immunological profiling for acute myocardial injury.

Figure. S4.

Discrimination of immunological profiling for acute kidney injury.

Tables

Table S1. Baseline clinical characteristics in patients with novel coronavirus pneumonia

| **Variable** | **Survial (n=136)** | **Death (n=27)** | ***P-value*** |
| --- | --- | --- | --- |
| Age, years | 57.46 ± 14.77 | 69.15 ± 13.12 | <0.001 |
| Males, n (%) | 72 (52.9) | 14 (51.9) | 0.542 |
| BMI, kg/m^2^ | 25.83 ± 2.91 | 25.66 ± 3.20 | 0.782 |
| Smoking, n (%) | 26 (19.1) | 3 (11.1) | 0.243 |
| COPD, n (%) | 14 (10.3) | 4 (14.8) | 0.345 |
| SBP, mmHg | 132.10 ± 19.43 | 126.33 ± 20.66 | 0.170 |
| DBP, mmHg | 78.28 ± 12.8 | 72.41 ± 11.77 | 0.030 |
| Heart rate, /min | 89.10 ± 18.43 | 82.36 ± 10.90 | 0.100 |
| Respiratory rate, /min | 20.93 ± 3.76 | 23.26 ± 3.68 | 0.004 |
| Severe type, n (%) | 40 (29.4) | 26 (96.3) | <0.001 |
| MODS, n (%) | 12 (8.8) | 13 (48.1) | <0.001 |
| **Laboratory findings** | | | |
| Hemoglobin, g/L | 128.04 ± 30.92 | 128.65 ± 22.45 | 0.924 |
| Platelet count, 10^9^/L | 239.01 ± 91.89 | 192.12 ± 133.19 | 0.029 |
| Hs-CRP, mg/L | 5.71 (4.31-6.13) | 13.73 (11.58-15.35) | <0.001 |
| Procalcitonin, ng/mL | 0.05 (0.03-0.09) | 0.17 (0.11-0.58) | <0.001 |
| NT-proBNP, pg/mL | 143 (49-387) | 659 (169-2854) | 0.001 |
| hs-CTn I, μg/mL | 0.00 (0.00-0.01) | 0.03 (0.01-0.12) | 0.003 |
| Albumin, g/L | 38.85 ± 11.77 | 34.37 ± 4.49 | 0.081 |
| Urea nitrogen, mmolL | 4.81 (3.70-6.30) | 8.43 (5.79-13.90) | <0.001 |
| Creatinine, μmolL | 61 (50-75) | 67 (48-104) | 0.217 |
| LDH, U/L | 297.04 ± 157.58 | 573.35 ± 277.28 | <0.001 |
| Fibrinogen, g/L | 3.89 ± 1.30 | 4.46 ± 2.25 | 0.104 |
| D-dimer, mg/L | 0.63 (0.32-3.31) | 3.48 (1.18-30.33) | <0.001 |
| PH | 7.41 ± 0.06 | 7.41 ± 0.07 | 0.929 |
| PO_2_, mmHg | 91.39 ± 45.54 | 54.33 ± 19.58 | <0.001 |
| PCO_2_, mmHg | 42.83 ± 7.77 | 39.62 ± 10.82 | 0.122 |
| Oxygen saturation, % | 92.4 ± 9.95 | 82.62 ± 12.28 | <0.001 |
| HCO_3_^-^, mmol/L | 26.97 ± 4.04 | 25.27 ± 7.03 | 0.144 |
| Lactate, mmol/L | 2.78 ± 1.25 | 2.52 ± 1.20 | 0.410 |

BMI, body mass index; COPD, chronic obstructive pulmonary disease; SBP, systolic blood pressure; DBP, diastolic blood pressure; MODS, multiple organ dysfunction syndrome; WBC, white blood cell count; Hs-CRP, high-sensitivity C-reactive protein; Hs-CTn I, high-sensitivity cardiac troponin I; NT-proBNP, N-terminal pro-brain natriuretic peptide; LDH, lactate dehydrogenase; PO_2_, partial pressure of oxygen; PCO_2_, partial pressure of carbon dioxide; HCO_3_^-^, bicarbonate.

Table S2. The incidence rates of mortality, multiple organ dysfunction syndrome (MODS) and severe pneumonia in patients with normal or abnormal immune situation

| **Outcomes** | **Normal** | **Abnormal** | ***P*-value** |
| --- | --- | --- | --- |
| **Cellular immunity** | | | |
| Mortality, % | 1 (2.0) | 26 (23.0) | <0.001 |
| MODS, % | 0 (0.0) | 25 (22.1) | <0.001 |
| Severe pneumonia, % | 4 (8.0) | 62 (54.9) | <0.001 |
| **Humoral immunity** | | | |
| Mortality, % | 13 (12.4) | 14 (24.1) | 0.045 |
| MODS, % | 15 (14.3) | 10 (17.2) | 0.387 |
| Severe pneumonia, % | 39 (37.1) | 27 (46.6) | 0.157 |

MODS, multiple organ dysfunction syndrome.

Table S3. Relationship between early immunity-related indexes and mortality in patients with novel coronavirus pneumonia

| **Variable** | **Survival (n=136)** | | |  | **Death (n=27)** | | | ***P*-value** |
| --- | --- | --- | --- | --- | --- | --- | --- | --- |
|  | M ± SD or | Down (%) | Up (%) |  | M ± SD or | Down (%) | Up (%) |  |
|  | M (25th-75th) |  |  |  | M (25th-75th) |  |  |  |
| WBC count, 10^9^/L | 5.83 (4.56-7.93) | 11 (8.1) | 23 (16.9) |  | 9.81 (6.23-11.90) | 2 (7.4) | 13 (48.1) | 0.001 |
| Neutrophil count, 10^9^/L | 3.74 (2.69-6.35) | 12 (8.8) | 33 (24.3) |  | 8.27 (6.23-10.76) | 2 (7.4) | 19 (70.4) | <0.001 |
| Neutrophil percentage, % | 69.06 ± 15.36 | 2 (1.5) | 44(32.4) |  | 83.95 ± 10.31 | 0 (0.0) | 21 (77.8) | <0.001 |
| Monocyte count, 10^9^/L | 0.47 (0.36-0.59) | 0 (0.0) | 33 (24.3) |  | 0.39 (0.24-0.61) | 0 (0.0) | 6 (22.2) | 0.821 |
| Monocyte percentage, % | 8.1 (5.2-10.4) | 10 (7.4) | 37 (27.2) |  | 4.2 (3.3-8.6) | 5(18.5) | 4 (14.8) | 0.002 |
| Basophil count, 109/L | 0.02 (0.01-0.03) | 0 (0.0) | 1(0.7) |  | 0.01(0.01-0.02) | 0 (0.0) | 0 (0.0) | <0.001 |
| Basophil percentage, % | 0.2 (0.2-0.4) | 0 (0.0) | 2(1.5) |  | 0.1(0.1-0.2) | 0 (0.0) | 0 (0.0) | 0.070 |
| Eosinophil count, 109/L | 0.02 (0.00-0.07) | 55 (40.4) | 1 (0.7) |  | 0.00 (0.00-0.01) | 22 (81.5) | 0 (0.0) | <0.001 |
| Eosinophil percentage, % | 0.5 (0.0-1.2) | 51 (37.5) | 43 (31.6) |  | 0.0 (0.0-0.1) | 24 (88.9) | 2 (7.4) | <0.001 |
| Lymphocyte percentage, % | 21.59 ± 12.40 | 57 (41.9) | 7 (5.1) |  | 8.85 ± 5.60 | 24 (88.9) | 0 (0.0) | <0.001 |
| Lymphocyte count, 10^9^/L | 1.22 ± 0.57 | 32 (23.5) | 0 (0.0) |  | 0.76 ± 0.51 | 17 (63.0) | 0 (0.0) | <0.001 |
| Immunoglobulin G, g/L | 12.94 ± 3.36 | 1 (0.7) | 15 (11.0) |  | 13.93 ± 3.98 | 0 (0.0) | 6 (22.2) | 0.25 |
| Immunoglobulin M, g/L | 0.99 ± 0.42 | 3 (2.2) | 1 (0.7) |  | 0.81 ± 0.31 | 2 (7.4) | 0 (0.0) | 0.07 |
| Complement 3, mg/dl | 102.78 ± 22.08 | 28 (20.6) | 0 (0.0) |  | 109.15 ± 21.36 | 4 (14.8) | 0 (0.0) | 0.241 |
| Complement 4, mg/dl | 23.73 ± 9.34 | 2 (1.5) | 4 (2.9) |  | 33.96 ± 13.61 | 0 (0.0) | 6 (22.2) | <0.001 |
| CD3^+^ T cell percentage, % | 66.20 ± 10.96 | 25 (18.4) | 0 (0.0) |  | 54.17 ± 11.86 | 13 (48.1) | 0 (0.0) | <0.001 |
| CD3^+^ T cell count, /μL | 753 (425-998) | 63 (46.3) | 0 (0.0) |  | 320 (226-486) | 25 (92.6) | 0 (0.0) | <0.001 |
| CD4^+^ T cell percentage, % | 39.58 ± 9.86 | 29 (21.3) | 4 (2.9) |  | 34.34 ± 8.99 | 12 (44.4) | 0 (0.0) | 0.013 |
| CD4^+^ T cell count, /μL | 460.25 ± 279.21 | 64 (47.1) | 0 (0.0) |  | 223.31 ± 94.04 | 26 (96.3) | 0 (0.0) | <0.001 |
| CD8^+^ T cell percentage, % | 24.57 ± 10.97 | 15 (11.0) | 9 (6.6) |  | 17.46 ± 8.26 | 11 (40.7) | 0 (0.0) | 0.002 |
| CD8^+^ T cell count, /μL | 244 (139-394) | 57 (41.9) | 0 (0.0) |  | 101 (53-155) | 24 (88.9) | 0 (0.0) | <0.001 |
| CD4^+^/CD8^+^ T cell | 1.78 (1.19-2.75) | 16 (11.8) | 57 (41.9) |  | 2.21 (1.39-3.43) | 2 (7.4) | 15 (55.6) | 0.123 |
| CD19^+^ B cell percentage, % | 14.57 (9.96-23.47) | 5 (3.7) | 36 (26.5) |  | 18.07 (10.90-23.51) | 0 (0.0) | 8 (29.6) | 0.361 |
| CD19^+^ B cell count, /μL | 130 (79-209) | 29 (21.3) | 2 (1.5) |  | 104 (67-162) | 7 (25.9) | 0 (0.0) | 0.095 |
| CD16^+^CD56^+^ NK cell percentage, % | 14.41 ± 7.70 | 6 (4.4) | 10 (7.4) |  | 24.03 ± 12.39 | 0 (0.0) | 11 (40.7) | <0.001 |
| CD16^+^CD56^+^ NK cell count, /μL | 115 (66-199) | 42 (30.9) | 0 (0.0) |  | 151 (74-199) | 8 (29.6) | 1 (3.7) | 0.516 |
| Hs-CRP, mg/L | 5.71 (4.31-6.13) | 0 (0.0) | 71(52.2) |  | 13.73 (11.58-15.35) | 0 (0.0) | 18(66.7) | <0.001 |
| Procalcitonin, ng/mL | 0.05 (0.03-0.09) | 0 (0.0) | 28(20.6) |  | 0.17 (0.11-0.58) | 0 (0.0) | 17(63.0) | <0.001 |

Reference value: leukocyte count, 3.5-9.5 * 10^9^/L; neutrophil count, 1.8-6.5 * 10^9^/L; neutrophil percentage, 40-75%; monocyte count, 0.1-0.6 * 10^9^/L; monocyte percentage, 3-10%; basophil count 0-0.06 * 10^9^/L; basophil percentage 0-1.0%; eosinophil count, 0.02-0.52 * 10^9^/L; eosinophil percentage 0.4-8.0%; lymphocyte percentage, 20-40%; lymphocyte count, 0.8-4.0 * 10^9^/L; immunoglobulin G, 7-16 g/L; immunoglobulin M, 0.4-2.3 g/L; complement 3, 0.9-1.8 mg/dl; complement 4, 0.1-0.4 mg/dl; CD3^+^ T cell percentage, 56-86%; CD3^+^ T cell count, 723-2737/μL; CD4^+^ T cell percentage, 33-58%; CD4^+^ T cell count, 404-1612/μL; CD8^+^ T cell percentage, 13-39%; CD8^+^ T cell count, 220-1129/μL; CD4^+^/CD8^+^ T cell, 0.9-2.0; CD19^+^ B cell percentage, 5-22%; CD19^+^ B cell count, 80-616/μL; CD16^+^CD56^+^ Natural killer (NK) cell percentage, 5-26%; CD16^+^CD56^+^ NK cell count, 84-724/μL; Hs-CRP (high-sensitivity C-reactive protein), 0-5 mg/L; procalcitonin, 0-0.1ng/mL.

Table S4. Logistic regression analysis regarding immunity-related indexes at admission for in-hospital mortality and multiple organ dysfunction syndrome (MODS).

| **Variable** | **Mortality** | |  | **MODS** | |
| --- | --- | --- | --- | --- | --- |
|  | **OR (95% CI)** | ***P*** |  | **OR (95% CI)** | ***P*** |
| **Leukocyte count, 10^9^/L** | | | | |  |
| Unadjusted | 1.130 (1.041-1.226) | 0.003 |  | 1.193 (1.079-1.318) | 0.001 |
| Adjusted | 1.144 (1.008-1.298) | 0.037 |  | 1.220 (1.082-1.375) | 0.001 |
| **Neutrophil count, 10^9^/L** | | |  |  |  |
| Unadjusted | 1.150 (1.057-1.252) | 0.001 |  | 1.212 (1.096-1.340) | <0.001 |
| Adjusted | 1.170 (1.024-1.337) | 0.021 |  | 1.230 (1.090-1.388) | 0.001 |
| **Neutrophil percentage, %** | |  |  |  |  |
| Unadjusted | 1.083 (1.041-1.126) | <0.001 |  | 1.103 (1.054-1.154) | <0.001 |
| Adjusted | 1.081 (1.020-1.147) | 0.009 |  | 1.116 (1.054-1.182) | <0.001 |
| **Monocyte count, 10^9^/L** | |  |  |  |  |
| Unadjusted | 1.143 (0.215-6.083) | 0.876 |  | 0.844 (0.144-4.935) | 0.851 |
| Adjusted | 1.241 (0.165-9.352) | 0.834 |  | 1.839 (0.305-11.083) | 0.506 |
| **Monocyte percentage, %** | |  |  |  |  |
| Unadjusted | 0.833 (0.730-0.950) | 0.006 |  | 0.747 (0.638-0.875) | <0.001 |
| Adjusted | 0.854 (0.702-1.038) | 0.112 |  | 0.766 (0.631-0.928) | 0.007 |
| **Basophil count, 10^7^/L** | |  |  |  |  |
| Unadjusted | 0.833 (0.612-1.135) | 0.247 |  | 0.660 (0.454-0.959) | 0.029 |
| Adjusted | 0.992 (0.680-1.448) | 0.967 |  | 0.584 (0.383-0.891) | 0.012 |
| **Basophil percentage, %** | |  |  |  |  |
| Unadjusted | 0.007 (0.001-0.172) | 0.002 |  | 0.032 (0.002-0.512) | 0.015 |
| Adjusted | 0.044 (0.001-1.58) | 0.087 |  | 0.008 (0.001-0.306) | 0.009 |
| **Eosinophil count, 10^7^/L** | |  |  |  |  |
| Unadjusted | 0.958 (0.887-1.035) | 0.274 |  | 0.968 (0.902-1.040) | 0.376 |
| Adjusted | 0.960 (0.860-1.073) | 0.472 |  | 0.955 (0.872-1.047) | 0.328 |
| **Eosinophil percentage, %** | |  |  |  |  |
| Unadjusted | 0.319 (0.125-0.813) | 0.017 |  | 0.509 (0.253-1.027) | 0.059 |
| Adjusted | 0.268 (0.060-1.189) | 0.083 |  | 0.557 (0.279-1.114) | 0.098 |
| **Lymphocyte percentage, %** | | | | | |
| Unadjusted | 0.877 (0.823-0.934) | <0.001 |  | 0.879 (0.826-0.937) | <0.001 |
| Adjusted | 0.842 (0.753-0.942) | 0.003 |  | 0.842 (0.764-0.928) | 0.001 |
| **Lymphocyte count, 10^9^/L** | | | | | |
| Unadjusted | 0.149 (0.052-0.429) | <0.001 |  | 0.213 (0.079-0.576) | 0.002 |
| Adjusted | 0.276 (0.075-1.019) | 0.053 |  | 0.361 (0.125-1.039) | 0.059 |
| **Immunoglobulin G,g/L** | | | | | |
| Unadjusted | 1.081 (0.947-1.234) | 0.250 |  | 1.033 (0.887-1.202) | 0.676 |
| Adjusted | 1.126 (0.922-1.375) | 0.243 |  | 0.965 (0.765-1.218) | 0.766 |
| **Immunoglobulin M, g/L** | | | | | |
| Unadjusted | 0.262 (0.061-1.131) | 0.073 |  | 0.722 (0.175-2.978) | 0.653 |
| Adjusted | 0.217 (0.022-2.091) | 0.186 |  | 1.514 (0.137-16.699) | 0.735 |
| **Complement 3, mg/dl** | | | | | |
| Unadjusted | 3.812 (0.41-35.464) | 0.240 |  | 1.024 (0.998-1.051) | 0.066 |
| Adjusted | 3.103 (0.103-93.081) | 0.514 |  | 1.045 (1.001-1.090) | 0.043 |
| **Complement 4, mg/dl** | | | | | |
| Unadjusted | 1.095 (1.038-1.156) | 0.001 |  | 1.108 (1.044-1.177) | 0.001 |
| Adjusted | 1.104 (1.010-1.208) | 0.03 |  | 1.197 (1.050-1.365) | 0.007 |
| **CD3^+^ percentage, %** | | | | | |
| Unadjusted | 0.919 (0.883-0.957) | <0.001 |  | 0.927 (0.891-0.964) | <0.001 |
| Adjusted | 0.931 (0.880-0.985) | 0.014 |  | 0.917 (0.872-0.965) | 0.001 |
| **CD3^+^ count T cell, /μL** | | | | | |
| Unadjusted | 0.996 (0.994-0.998) | <0.001 |  | 0.997 (0.996-0.999) | 0.001 |
| Adjusted | 0.996 (0.994-0.999) | 0.004 |  | 0.997 (0.996-0.999) | 0.009 |
| **CD4^+^ percentage, %** | | | | | |
| Unadjusted | 0.947 (0.906-0.990) | 0.016 |  | 0.924 (0.880-0.970) | 0.001 |
| Adjusted | 0.953 (0.886-1.024) | 0.191 |  | 0.916 (0.861-0.975) | 0.006 |
| **CD4^+^ count T cell, /μL** | | | | | |
| Unadjusted | 0.994 (0.991-0.997) | <0.001 |  | 0.995 (0.992-0.997) | <0.001 |
| Adjusted | 0.994 (0.989-0.998) | 0.009 |  | 0.994 (0.991-0.998) | 0.003 |
| **CD8^+^ percentage T cell, %** | | | | | |
| Unadjusted | 0.908 (0.855-0.963) | 0.001 |  | 0.982 (0.939-1.027) | 0.432 |
| Adjusted | 0.883 (0.802-0.973) | 0.012 |  | 0.971 (0.913-1.033) | 0.355 |
| **CD8^+^ count T cell, /μL** | | | | | |
| Unadjusted | 0.991 (0.986-0.995) | <0.001 |  | 0.996 (0.992-0.999) | 0.013 |
| Adjusted | 0.993 (0.988-0.998) | 0.008 |  | 0.996 (0.993-1.000) | 0.077 |
| **CD4^+^/CD8^+^ T cell** | | | | | |
| Unadjusted | 0.991 (0.944-1.041) | 0.728 |  | 0.874 (0.592-1.290) | 0.499 |
| Adjusted | 1.035 (0.905-1.185) | 0.612 |  | 0.808 (0.497-1.313) | 0.389 |
| **CD19^+^ B cell percentage, %** | | | | | |
| Unadjusted | 1.027 (0.984-1.073) | 0.222 |  | 1.023 (0.979-1.069) | 0.316 |
| Adjusted | 1.061 (1.001-1.124) | 0.048 |  | 1.033 (0.981-1.088) | 0.219 |
| **CD19^+^ B cell count, /μL** | | | | | |
| Unadjusted | 0.995 (0.990-1.000) | 0.061 |  | 0.995 (0.990-1.000) | 0.075 |
| Adjusted | 0.998 (0.992-1.004) | 0.502 |  | 0.997 (0.990-1.003) | 0.276 |
| **CD16^+^CD56^+^ NK cell percentage, %** | | | | | |
| Unadjusted | 1.106 (1.054-1.160) | <0.001 |  | 1.065 (1.020-1.112) | 0.004 |
| Adjusted | 1.001 (0.996-1.005) | 0.779 |  | 1.074 (1.007-1.144) | 0.030 |
| **CD16^+^CD56^+^ NK cell count, /μL** | | | | | |
| Unadjusted | 1.002 (0.999-1.005) | 0.183 |  | 1.001 (0.998-1.005) | 0.368 |
| Adjusted | 1.001 (0.996-1.005) | 0.779 |  | 1.001 (0.998-1.005) | 0.481 |
| **High-sensitivity C-reactive protein, mg/L** | |  |  |  |  |
| Unadjusted | 1.018 (1.009-1.027) | <0.001 |  | 1.020 (1.010-1.030) | <0.001 |
| Adjusted | 1.024 (1.008-1.040) | 0.004 |  | 1.025 (1.011-1.039) | <0.001 |
| **Procalcitonin, ng/mL** | |  |  |  |  |
| Unadjusted | 8.126 (2.318-28.494) | 0.001 |  | 85.26 (4.84-1501.80) | 0.002 |
| Adjusted | 2.981 (0.456-19.509) | 0.254 |  | 211.87 (5.45-8231.81) | 0.004 |

Variables adjusted by age, gender, body mass index, smoke, systolic blood pressure, heart rate, and MODS for in-hospital mortality. Variables adjusted by age, gender, body mass index, smoke, systolic blood pressure, and heart rate for MODS. OR, odds ratio; CI, confidence interval.
