# Supplementary figures and images for: Immune dysfunction leads to mortality and organ injury in patients with COVID-19 in China: insights from ERS-COVID-19 study
Source: Signal Transduct Target Ther. 2020 May 5;5:62. doi: 10.1038/s41392-020-0163-5 (PMC7198844; doi:10.1038/s41392-020-0163-5)

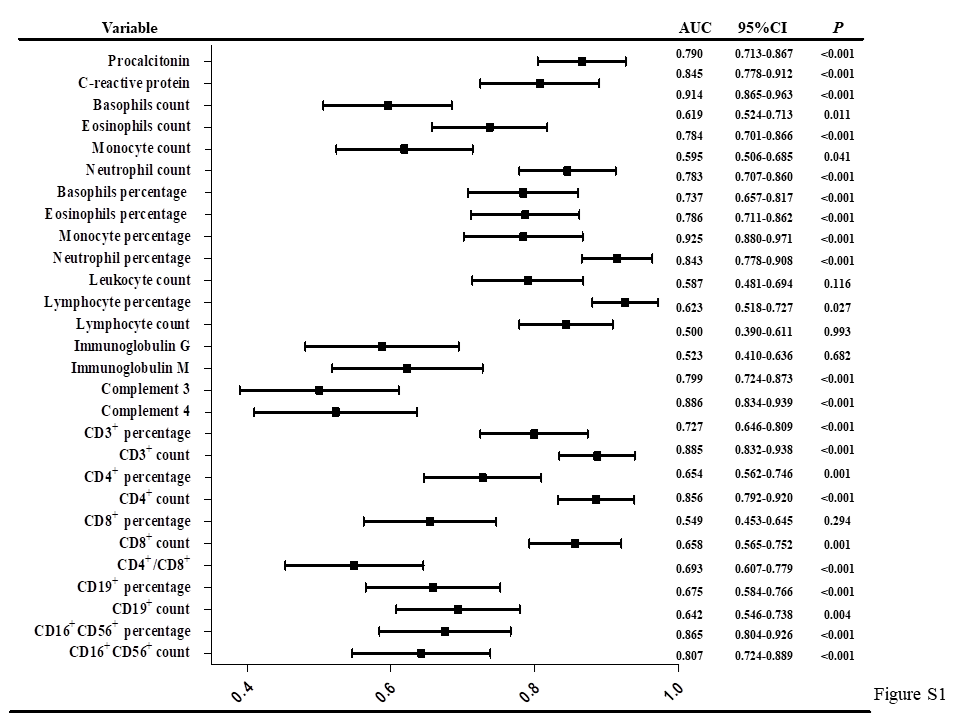

Supplement: Supplementary file 2 — Figure S1 [file 41392_2020_163_MOESM2_ESM.tif]

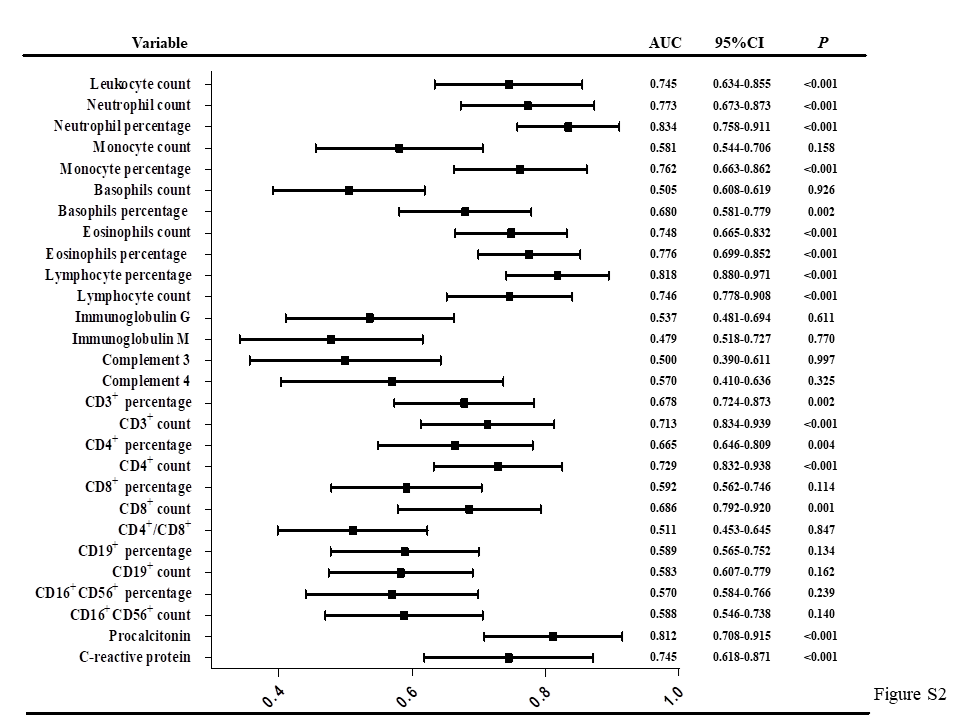

Supplement: Supplementary file 3 — Figure S2 [file 41392_2020_163_MOESM3_ESM.tif]

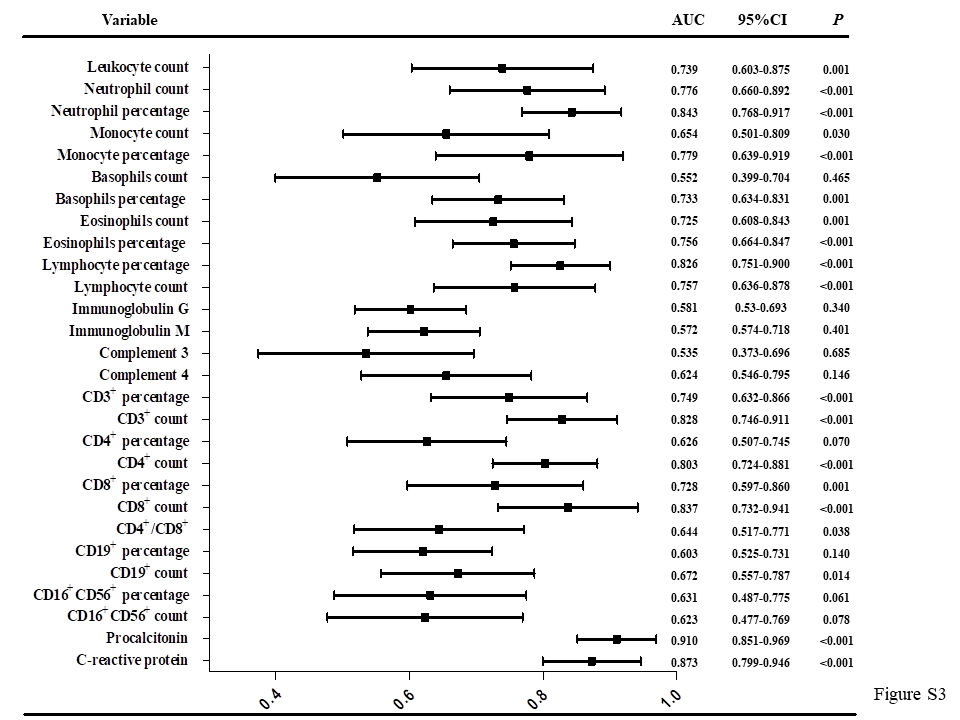

Supplement: Supplementary file 4 — Figure S3 [file 41392_2020_163_MOESM4_ESM.tif]

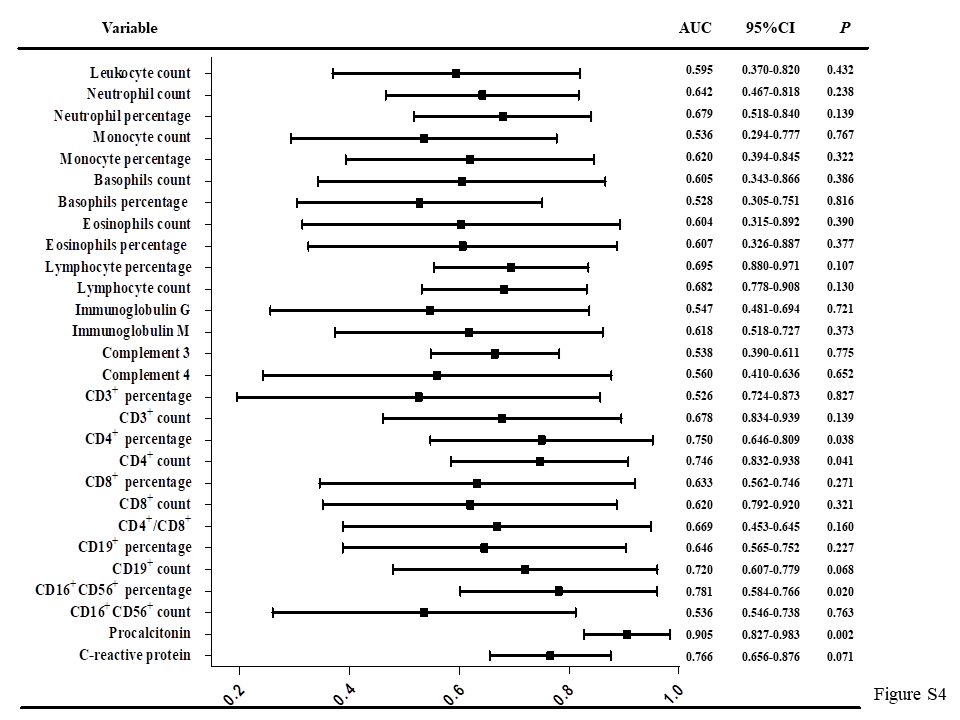

Supplement: Supplementary file 5 — Figure S4 [file 41392_2020_163_MOESM5_ESM.tif]
